# The last strategy for re-dissemination of HPV vaccination in Japan while still under the suspension of the governmental recommendation

**DOI:** 10.1038/s41598-020-73120-1

**Published:** 2020-09-30

**Authors:** Yutaka Ueda, Asami Yagi, Hazuki Abe, Satoshi Nakagawa, Ryoko Minekawa, Haruo Kuroki, Ayako Miwa, Tadashi Kimura

**Affiliations:** 1 Department of Obstetrics and Gynecology, Osaka University Graduate School of Medicine, 2-2, Yamadaoka Suita, Osaka, 567-0871 Japan; 2 Ogata Family Clinic, Ashiya, Hyogo Japan; 3 Sotobou Kodomo Clinic, Isumi, Chiba Japan; 4 Department of Obstetrics and Gynecology, Juntendo University, Tokyo, Japan

**Keywords:** Health care, Oncology

## Abstract

In Japan, the governmental recommnendation of HPV vaccine has been suspended since June 2013, due to media reports of alleged adverse vaccination events. Although evidence of effectiveness and safety of the HPV vaccine has been universally demonstrated, and the medical and academic organizations across Japan have requested the resumption of the government’s recommendation, the Japanese government has not changed their official stance towards the HPV vaccine. Under the current suspension of the national government’s recommendation, one local government Isumi City started sending a leaflet containing information of cervical cancer and HPV vaccine, but not recommendation for the vaccine, to the tagted girls born in the fiscal year (FY) 2003. The cumulative vaccination rate of them reached 10.07% (14/139), which was significantly higher than that (0.00%) for girls born in FY 2002 who did not receive such a leaflet (*p* < 0.001). We sincerely ask the national government to change their stance towards the HPV vaccine. We also strongly suggest that, in the meantime, local governments immediately begin to provide an appropriate information of cervical cancer and HPV vaccine to the targeted girls and their parents in a way similar to what Isumi City has now shown to be effective.

## Introduction

The majority of cervical cancer cases can be easily preventable by a combination of HPV vaccination and routine cervical cancer screening. Despite this fact, we have previously shown that the age-adjusted incidence of cervical cancer in Japan has been steadily increasing since 2000^1^. The cervical cancer screening rate in Japan has become extremely low, as compared to other developed countries over the same period. The screening rate for young women at the age of 20–24 is currently only around 10%, and even in females aged 25–29 and 30–39, the screening rate is only around 10–20% and around 10–30%, respectively^2^. Low inoculation rate of HPV vaccine is another issue to overcome to reduce cervical cancer incidence.

Financial support from the Japanese government for HPV vaccination for girls aged 13–16 began in the fiscal year (FY: April–March) 2010^3^. Females born between FY 1994 and FY 1999 have enjoyed rates of vaccination protection approaching 70%, although the vaccination was not free^4–6^. In April 2013, HPV vaccination became a part of the routine immunization program, meaning that thereafter females aged 12–16 could receive the vaccination for free. News stories related to cervical cancer prevention such as risk of developing cervical cancer, its etiology, and the effects of vaccination frequently appeared in the media until 2012, however, after March 2013 they were replaced with anti-vaccination contents^7^. Due to repeated media reports of alleged adverse vaccination events, the Ministry of Health, Labour and Welfare (MHLW) suspended its recommendation for the HPV vaccine in June 2013. For girls born in or after 2000 in Japan, who reached the age for vaccination after suspension of the governmental recommendation, their HPV vaccination rates have been declined, despite the vaccination was still free^3^. The cumulative vaccination rates for girls born in FY2000, FY2001, FY 2002 and FY2003 were reported to be 14.3%, 1.6%, 0.4% and 0.2%, respectively^8^. In Japan, parental consent is required for their daughter’s HPV vaccination, and our previous internet survey suggested that acceptance or refusal of the HPV vaccine was determined predominantly by the mother's perceptions of risk versus benefits, rather than the daughter's wishes in Japan^9^.

Protective effect of HPV vaccine against cervical cancer or its precancerous lesion of the cervix has been shown not only from overseas^10^ but also in Japan^11,12^, and safety of HPV vaccine has been also internationally and domestically demonstrated^13,14^. The medical and academic organizations across Japan have requested the resumption of the government’s recommendation;^15^ nevertheless the MHLW has not changed their official stance towards the HPV vaccine. Although the HPV vaccine is still regarded as a ‘routine vaccination’ in Japan, the national government still does not ‘officially recommend’ it. In addition, neither more potent 9-valent HPV vaccine nor immunization for boys have been approved yet in a national routine immunization program^3,16^. Moreover, seven years have now passed since the announcement of the governmental suspension of the HPV vaccine recommendation. We have already reported that Japanese mothers’ intentions to have their daughters vaccinated have been further eroded over this period^17^.

Under these dire circumstances, we must find a way to re-disseminate HPV vaccination nationwide as a countermeasure against the recent increase of cervical cancer among young women^1^. A recent survey conducted by the MHLW found that only 5.6% of all local governments across the nation (97/1741), under the current suspension of the government’s recommendation, sent leaflets containing only information of HPV vaccine, but not recommendation for the vaccine, individually to the girls and their parents^18^. In the present study, we have analyzed the effectiveness of one type of leaflet that one local government, not on a national basis, has been sending to individuals of HPV vaccination age or to their parents under the governmental suspension of the HPV vaccine recommendation.

## Methods

Isumi City is a small municipality, with a population of 38,000 (Table 1)^19^. On July 29, 2019, the Isumi City government started sending an informational/educational leaflet (Table 2 and Supplementary Figure), which addressed the risks of cervical cancer and the role of the HPV vaccine in its prevention, to all 139 girls who were born in FY 2003 and reached 16 years old at that time. The present study was a population-based analysis to compare the first vaccination rates before and after this project, and necessary data on monthly vaccination numbers of girls in the targeted age group was provided by the Division of Health Promotion and Senior Services, Isumi City.

**Table 1:** Characteristics of the Isumi City (as of January 2019).

	Isumi City	Japan
Population	38,242	127,443,563
Proportion of those aged less than 15	9.04%	12.37%
Proportion of those aged 15–64	51.38%	60.03%
Proportion of those aged 65 and older	39.58%	27.61%

Ministry of Internal Affairs and Communications^19^.

https://www.soumu.go.jp/main_content/000633314.pdf#search=%27%E7%94%9F%E7%94%A3%E5%B9%B4%E9%BD%A2%E4%BA%BA%E4%BD%8F%E6%B0%91+%E4%BA%BA%E5%8F%A3+%E4%B8%96%E5%B8%AF%E6%95%B0%27.

**Table 2:** Main contents of the leaflet.

Cervical cancer is caused by HPV, which is transmitted through sexual intercourse
In Japan, approximately 10,000 women are newly diagnosed with cervical cancer and around 3000 women die from cervical cancer each year
Cervical cancer has been increasing in women in their 20 s and 30 s, the peak ages of pregnancy and delivery
HPV vaccine is effective for preventing infection with HPV-16 and 18, which are responsible for around 65% of cervical cancer
The target age for routine HPV vaccination for girls is 12–16 years
The most common adverse events after HPV vaccination are pain, redness, and swelling at the inoculation site
Fainting can occur due to various stimuli such as pain, fear, and excitement caused by the injection
WHO confirmed effectiveness and safety of HPV vaccine, and stated that policy decisions based on weak evidence, leading to lack of use of safe and effective vaccines, can result in real harm, referring to the situation in Japan
In the event of a serious adverse events after vaccination, compensation is available under the Immunization Act
The recommendations for HPV vaccination from the national government has been suspending, but those who wish to be inoculated and are eligible for routine vaccination can receive the vaccine free of charge
Regular cervical cancer screenings are required after age 20

Written informed consent was obtained to use their personal data regarding HPV vaccination for the girls who got immunized in FY 2019. There was only one person whose informed consent was not obtained and excluded from the analysis. In contrast, for the girls who got immunized by FY 2018, informed consent was obtained through opt-out methods. It was shown as < 10 if the number of girls who was vaccinated is less than 10 before and in FY 2018, in view of not revealing personal information based on the ethical regulation for epidemiological research in Japan. Likewise, the total number of targeted girls in each FY was also withheld, so that each girl is not to be identified personally.

### Statistics

The rates of HPV vaccination were compared by Fisher’s exact test. The level of statistical significance was set at *p* = 0.05.

### Ethical statement

This study was approved by the Institutional Review Board and Ethics Committee of the Osaka University Medical Hospital (#13261-9). We conducted the present study in accordance with relevant guidelines and regulations.

### Statement of significance

Under the current suspension of the national government’s recommendation of HPV vaccine, providing an appropriate information of cervical cancer and HPV vaccine to the targeted girls and their parents from a local government was shown to be effective.

## Results

When the Japanese government was actively promoting the vaccine, the cumulative rates of the first (of three) vaccination shots in the targeted girls born between FY 1994 and FY 1999 substantially increased from 58.60 to 94.67% (Table 3). However, beginning in 2013, when the government withdrew its recommenadtion, the vaccination rates in Isumi City dropped dramatically, and none of the girls born in FY 2001 or 2002 received vaccination by the beginning of FY 2019, although the HPV vaccine was still regarded in Japan as a ‘routine vaccination’ and people can be immunized in medical facilities on request at any time.

**Table 3:** Number of the first vaccination shots received by targeted girls, by birth FY.

		Vaccination FY												Vaccination rate
		2010	2011	2012	2013	2014	2015	2016	2017	2018	2019 April–July	2019 Aug–Dec		
Birth FY	1994	< 10*	107											58.60%
	1995	108	31											88.53%
	1996	100	42	< 10*										85.14%
	1997	117	22	< 10*	< 10*									85.63%
	1998	132	< 10*	< 10*	0	0								94.67%
	1999			116	< 10*	0	0							74.52%
	2000			0	41	0	0	< 10*						25.45%
	2001				0	0	0	0	0					0.00%
	2002					0	0	0	0	0				0.00%
	**2003**						0	0	0	0	2**	12**		10.07%
	2004							0	0	0	1**	0		0.76%
	2005								0	0	0	1**		0.96%
	2006									0	2**	2**		3.10%
	2007										0	0		0.00%

Bold value indicates the girls receiving informational/educational leaflets.

*For the girls who got vaccinated by FY2018.

For the girls who got immunized by FY 2018, informed consent was obtained through opt-out methods. It was shown as < 10 if the number of girls who was vaccinated is less than 10 before and in FY 2018, in view of not revealing personal information based on the ethical regulation for epidemiological research in Japan. Likewise, the total number of targeted girls in each FY was also withheld, so that each girl is not to be identified personally.

**For the girls who got vaccinated in FY2019.

Written informed consent was obtained to use their personal data regarding HPV vaccination for the girls who got immunized in FY 2019. There was only one person whose informed consent was not obtained and excluded from the analysis.

*FY* fiscal year (April–March).

**Figure 1:**
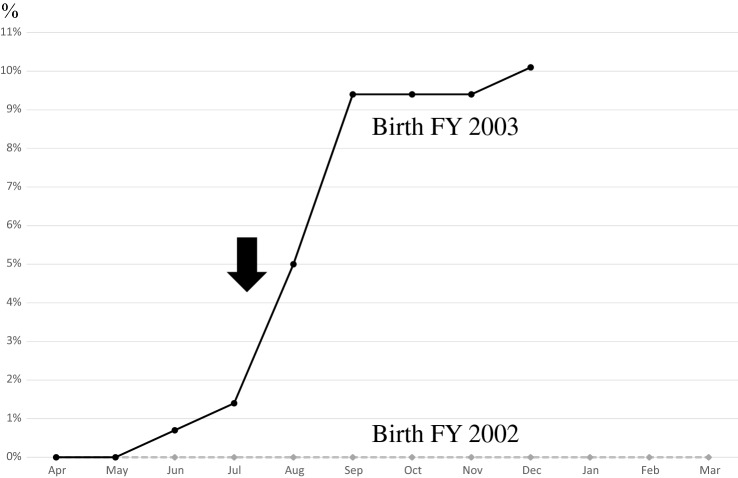
Cumulative rate of first vaccinations of targeted girls aged 16 born in FY 2002 and 2003. Solid line: born in FY 2003. Dotted line: born in FY 2002. Black arrow: Individual notification was sent from the local government of Isumi City on July 29, 2019).

In response, the Isumi City government started on July 29, 2019 sending an informational/educational leaflet which explained the risks of cervical cancer and role the HPV vaccine in its prevention, to all 139 girls who were born in FY 2003 and reached 16 years old at that time. FY 2019 was the last one when those girls could get HPV vaccination. Only 2 girls born in FY 2003 in the city had been vaccinated prior to the individual leaflet/notification. After receiving the leaflet, 12 girls got immunized (Fig. 1). The cumulative vaccination rate significantly increased from 1.44 (2/139) to 10.07% (14/139) (*p* = 0.003). Subsequently, the cumulative vaccination rate for girls born in FY 2003 in Isumi City reached 10.07% (14/139) by December 31, 2019, which was significantly higher than that (0.00%) for girls born in FY 2002 who did not receive such a leaflet (*p* < 0.001) (Table 3).

On the other hand, out of 489 girls born later in FY 2004–2007, who were also the targets of HPV vaccine but did not receive the individual notification/leaflet, only 6 girls had been vaccinated by December 31, 2019. This proportion 1.23% (6/489) for those born in FY 2004–2007 was significantly smaller than the rate 10.07% for the girls born in FY 2003 who received the leaflet (14/139) (*p* < 0.001), while it did not show significant difference from the rate 0.00% (0/148) for the girls born in FY 2002 (*p* = 0.34). Moreover, in 2019, 3 girls born in FY 2004–2007 received the HPV vaccine between April and July without information leaflet, and another 3 girls received it between August and December, however the cumulative vaccination rate did not increase significantly throughout the year (3/489 to 6/489, *p* = 0.51).

These results indicated that increase in the HPV vaccination rate among the targeted girls born in FY 2003 was strongly influenced by the leaflet sent individually by the local government, as long as no other factors, such as mothers’ change in awareness for vaccination for their daughters, seemed to make impact in our analysis.

## Discussion

The World Health Organization (WHO) regarded vaccine hesitancy, the reluctance or refusal to vaccinate despite the availability of vaccines, as one of the 10 threats to global health in 2019^20^. Mechanisms of HPV vaccine hesitancy after the suspension of the governmental recommendation at first were explained from the viewpoints of behavioral economics^21^. Lack of information on HPV vaccine in the targeted girls and their parents has become an additional barrier because only 5.6% of all the local governments across the nation sent certain types of leaflets containing information on HPV vaccine individually to the girls and their parents inside the municipality under the suspension of the governmental recommendation^18^. Any promotional activities of HPV vaccine have not been conducted to increase HPV vaccination rates by local governments, doctors, and school teachers, because of suspension.

Sending individually an information leaflet about cervical cancer and HPV vaccination from a local government to the targeted girls and their parents appeared to be effective for promoting HPV vaccination. By the first of FY2020, females born in FYs 2000, 2001, 2002 and 2003 were already over 12–16, the targeted ages for the vaccine. Future risk of cervical cancer for girls born in FY 2004 will significantly increase in FY 2020 when they become 16 years old, which is the last year for routine HPV vaccination^22,23^, unless the government's recommendations for HPV vaccination are reinstated. We sincerely ask the national government to change their stance towards the HPV vaccine. We also strongly suggest that, in the meantime, local governments could immediately begin, even if it is still under the current circumstances of the suspension of the governmental recommendation, to provide an appropriate information of cervical cancer and HPV vaccine to the targeted girls and their parents in such a way that Isumi City has now proven it useful. Up until now, there have not been any other reports to demonstrate the effectiveness of sending an information leaflet by local governments in Japan.

A previous internet survey conducted by the MHLW found that 38.8% of the tagted girls had not heard of significance and safety of HPV vaccine at all, and that 33.8% of the mothers of the targeted girls wanted to get appropreate information at the consultation counter of a local government^17^. Moreover, 45.0% of the targeted girls and 38.4% of thier mothers replied that they could not decide whether to receive the HPV vaccine because they did not have adequate knowledge about HPV vaccine well. In Japan, mothers play more important roles for decision of their daughters’ vaccination^9^. Fathers' participation in the mothers' decision-making did not increase the likelihood of HPV vaccination for their daughters, however, an educational leaflet was proved to be effective to enhance fathers’ enrollment for decision of their daughters’ vaccination^24^.

We believe that providing an appropriate information of cervical cancer and HPV vaccine to the targeted girls and their parents, which can be conducted under the current circumstances, is a potentially effective tool to support decision-making of HPV vaccination for the targetted girls and their mothers, and it is educational as well for their knowledge and attitudes toward cervical cancer. It was demonstrated that effectiveness was enhanced when doctors explained about HPV vaccine using with informational leaflet, probably due to proper opportunities to understand the content well and ask questions^25^. Improvement of the contents of leaflets by taking viewpoints of behavioral economics might further increase HPV vaccination rates^21^. There was a report that secondary acceptance of HPV vaccination by the parents of adolescents is more common than we might suppose^26^. We could improve vaccination rate more if we consider this possibility and repeat providing the leaflets. We suppose that this is the last strategy for re-dissemination of HPV vaccination in Japan while still under the suspension of the governmental recommendation We will no longer overlook this situation.

## Supplementary information

Supplementary Figure.

## Abbreviations

FY: Fiscal year
HPV: Human papillomavirus
MHLW: Ministry of Health, Labour and Welfare
WHO: World Health Organization
