# Supplementary figures and images for: The last strategy for re-dissemination of HPV vaccination in Japan while still under the suspension of the governmental recommendation
Source: Sci Rep. 2020 Sep 30;10:16091. doi: 10.1038/s41598-020-73120-1 (PMC7527341; doi:10.1038/s41598-020-73120-1)

## Slide 1
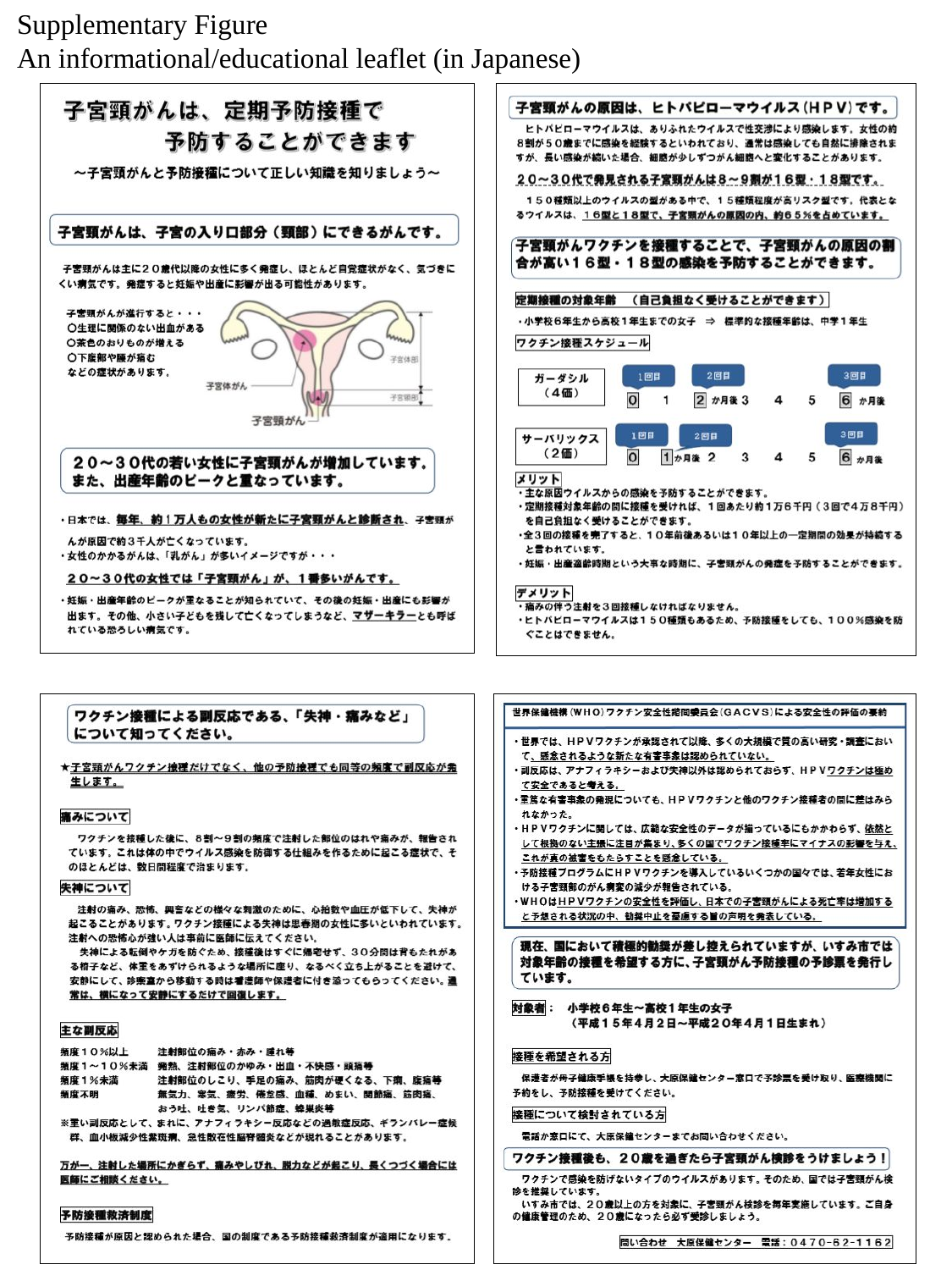

Supplementary Figure
An informational/educational leaflet (in Japanese)

Supplement: Supplementary file 1 — Supplementary Figure. [file 41598_2020_73120_MOESM1_ESM.pptx]
